# Nitrogen-Activated CLV3/ESR-Related 4 (CLE4) Regulates Shoot, Root, and Stolon Growth in Potato

**DOI:** 10.3390/plants12193468

**Published:** 2023-10-03

**Authors:** Maria S. Gancheva, Lyudmila A. Lutova

**Affiliations:** Department of Genetics and Biotechnology, Saint Petersburg State University, Universitetskaya emb. 7/9, Saint Petersburg 199034, Russia

**Keywords:** potato, CLE peptide, CLE4, NIN-like proteins (NLPs), nitrate, nitrate-responsive elements (NREs), shoot apical meristem (SAM), Identity of Tuber1 (IT1), tuber

## Abstract

In potato, high levels of nitrogen (N) can lead to excessive vegetative growth at the expense of tuber development, resulting in lower yield and poor-quality tubers. We found that *Solanum tuberosum CLE4* (*StCLE4*) is expressed most strongly in the roots grown in N-rich media, and it positively regulates potato root growth under N-deficient conditions. We noted that StCLE4 functions as a negative regulator of normal shoot apex development similar to CLV3 in Arabidopsis. Transcriptomic analysis revealed that overexpression of *StCLE4* resulted in the repression of the *StIT1* gene, a regulator of potato tuber initiation. *StCLE4*-overexpressing stolons were converted into branches, that were similar to a mild phenotype of the *it1* (*identity of tuber 1*) mutant. We also found that NIN-like proteins, key regulators of nitrate signaling bind to the regulatory sequence of *StIT1* in a yeast one-hybrid assay. Taken together, our findings suggest that StCLE4 regulates shoot, root, and stolon growth in potato.

## 1. Introduction

The CLAVATA3/EMBRYO SURROUNDING REGION (CLE) peptides are a group of hormones that play a crucial role in plant development and growth. Among them, CLV3 regulates the size of the shoot apical meristem (SAM) by downregulating the expression of the *WUSCHEL* (*WUS*) gene encoding a homeodomain transcription factor. Other *CLE* genes, including *CLE1*, *CLE4*, *CLE6*, and *CLE7*, can complement the enlarged meristem of the *clv3* mutants when they are expressed under the control of the *CLV3* promoter [1,2]. At the same time, the *CLE1*, *CLE3*, *CLE4*, and *CLE7* genes are induced in roots under nitrogen-deficient conditions, and they regulate root growth and branching in response to nitrogen availability [3]. *CLE* genes have been identified in various plant species, including *Arabidopsis thaliana*, rice, and maize. Previously we have identified *CLE* genes in potato, *Solanum tuberosum* L. (*StCLE*) and found that the expression level of several *CLE* genes is regulated by nitrogen supply [4]. Nitrogen is a macronutrient that is essential for plant growth and development, and it is often a limiting factor in crop production. Adequate nitrogen supply is necessary for the formation of healthy and high-yielding potato tubers. During the early stages of tuber development, nitrogen regulates the formation of stolons, which are stem-like structures that develop from the lower nodes of the potato plant. As tuber development progresses, nitrogen is required for the accumulation of starch and other nutrients in the tubers. Nitrogen deficiency during this stage can result in reduced tuber size and lower yield. However, excessive nitrogen supply can also have negative effects on tuber development. Excessive nitrogen levels can cause an overabundance of vegetative growth, which can have a negative impact on tuber development, ultimately resulting in lower yields and lower quality tubers. Therefore, it is important to maintain a balanced nitrogen supply throughout the growing season to ensure optimal tuber development in potato plants.

We previously found that the *StCLE4* gene was induced in potato roots under N-rich growth conditions and suggested that StCLE4 could be a negative regulator of tuberization. In this study, we show that the *StCLE4* promoter drives GUS reporter activity in the roots grown in N-rich media. Plants with the overexpression of *StCLE4* demonstrated a *wus*-like phenotype, with the arrested growth of the shoot apical meristem (SAM). The overexpression of *StCLE4* also resulted in the stimulation of root growth and non-swelling stolons that were converted into branches. The transcriptomic studies of *StCLE4*-overexpressing plants revealed the repression of the *IDENTITY OF TUBER1* (*IT1*) gene, a regulator of potato tuber initiation. Overall, overexpression phenotype and RNA-Seq expression profiles suggest the roles of StCLE4 in the regulation of shoot, stolon, and root growth. 

## 2. Results

### 2.1. Promoter Activity of StCLE4 Gene in Potato Roots

**Figure 1:**
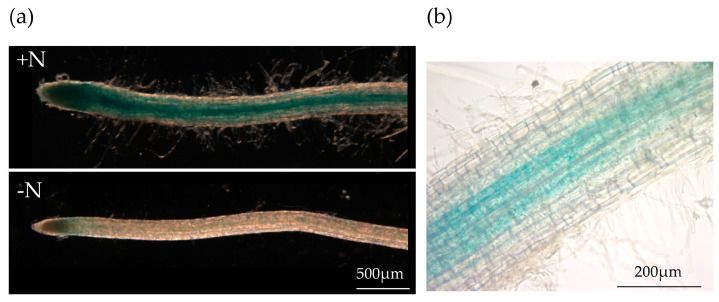
*promStCLE4:GUS* expression in potato roots. (**a**) The *promStCLE4*-driven GUS reporter is more active in the roots grown in N-rich media (+N). (**b**) The *StCLE4* promoter activity in the vascular tissues of the root.

The *StCLE4* gene belongs to the same clade of *CLE*s as the nitrate-regulated *CLE* genes of *A. thaliana* (*AtCLE1*, *-3*, *-4*, and *-7*) [4] that are induced upon N deprivation and suppress the initiation of lateral roots [3]. Previously, we evaluated the expression of the *StCLE4* gene in the roots of potato exposed to different N-availability conditions using RT-qPCR. Opposite to *A. thaliana CLE* genes, the StCLE4 gene was positively regulated by nitrogen supply [4]. In this study, to determine the expression pattern of *StCLE4* in potato roots, we generated *promCLE4:GUS* fusion constructs using 2963 bp of the genomic region upstream of *StCLE4*. The *StCLE4* promoter drove GUS activity in the vascular tissues of the root (Figure 1b), and the reporter gene was expressed most strongly on N-rich media (Figure 1a), confirming that this gene is activated under high nitrogen conditions. 

### 2.2. Effects of Overexpressing StCLE4 and StCLE4_G6T_ Genes on Root, Shoot, and Stolon Growth 

**Figure 2:**
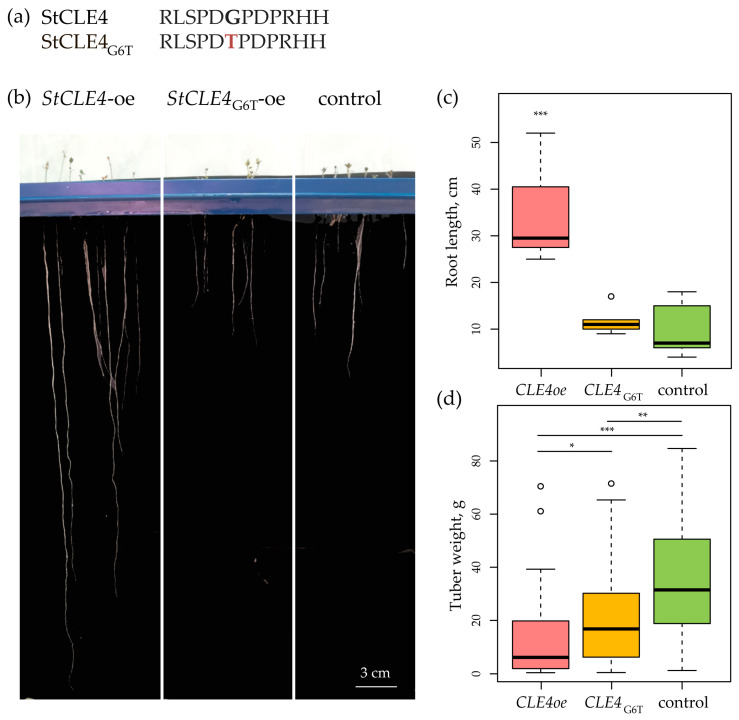
(**a**) Native StCLE4 peptide and amino acid-substituted StCLE4_G6T_ peptide. (**b**–**d**) Effects of *StCLE4* and *StCLE4_G6T_* overexpression on root growth in aeroponic nutrient medium without nitrogen (**b**,**c**) and on tuber weight (**d**). *, *p*-value < 0.05; **, *p*-value < 0.01; ***, *p*-value < 0.001.

To further investigate the functions of StCLE4, we used antagonistic peptide technology [5]. Specifically, we created an antagonistic *StCLE4* construct by mutating a codon encoding glycine in the CLE motif-coding region (which is known to play a crucial role in the biological activity of CLE) to threonine-coding codon (G6T) (Figure 2a). Vectors carrying the *StCLE4* and *StCLE4_G6T_* genes under the constitutive *35S* promoter were obtained. The overexpression of *StCLE4* resulted in the stimulation of root growth in aeroponic nutrient medium without nitrogen (Figure 2b,c) and the reduction in tuber weight (Figure 2d). At the same time, we did not detect any alterations in the growth of *StCLE4_G6T_*-overexpressing potato plants, except lower tuber weight (Figure 2 and Figures S1 and S2).

**Figure 3:**
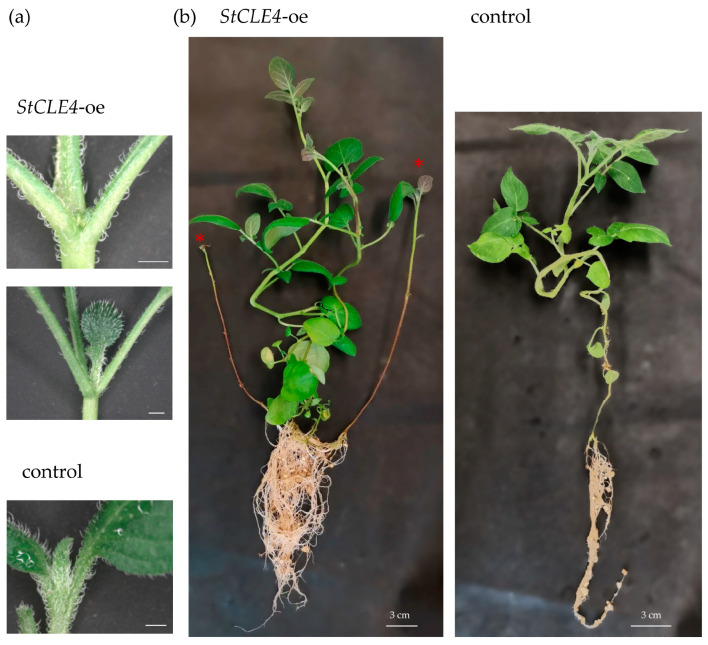
Effects of *StCLE4* overexpression (*StCLE4*-oe) on apex growth of three-week-old plants (**a**) and stolon growth of two-month-old plants (**b**) that were grown in a growth chamber under long days (16 h light/8 h dark and 22 °C). Non-swelling stolons that transformed into branches are marked by a red asterisk. Scale bar in panel (**a**) = 2 mm.

The overexpression of *StCLE4* also demonstrated a phenotype similar to the phenotypes described for the overexpression of *CLV3*, *CLE2*, *-3*, *-4*, *-5*, *-6*, *-7*, *-9*, *-10*, *-11*, and *-13* genes in *A. thaliana* [6]. The observed phenotypes were characterized by the arrest of leaf development from the SAM and subsequent recovery via the activation of axillary buds (Figure 3a), which is very similar to *wus* loss-of-function mutants [7]. In contrast to Arabidopsis plants overexpressing *CLV3*, *CLE9*, *-10*, *-11*, and *-13*, all potato plants overexpressing *StCLE4* resumed growth after the initial SAM arrest (Figure 3a). Moreover, non-swelling stolons that converted into aerial shoot branches were observed on plants overexpressing *StCLE4* (Figure 3b and Figure S2). 

### 2.3. RNA-Sequencing of Transgenic Potato Leaves and Roots with StCLE4 and StCLE4_G6T_ Overexpression 

To study the effect of gene overexpression on the potato transcriptome, we performed RNA sequencing analysis of the leaves and roots of transgenic potato plants overexpressing the *StCLE4* and *StCLE4_G6T_* genes. RNA was isolated from the plants grown in both nitrogen-rich and nitrogen-free media. The obtained RNA-seq data were analyzed using bioinformatics tools to identify differentially expressed genes (DEGs) between the transgenic and control plants. A total of 1096 DEGs were detected in the leaves, and 384 DEGs were detected in the roots of control and *StCLE4*-overexpressing plants grown in nitrogen-free media (Table S1, Figure S3). In total, 1942 DEGs were detected in the leaves and 391 DEGs in the roots of control and *StCLE4_G6T_*-overexpressing plants grown on nitrogen-free media (Table S1).

Gene ontology analysis was performed to identify the biological processes affected by *StCLE4* and *StCLE4_G6T_* overexpression. The results showed that the overexpression of the *StCLE4* and *StCLE4_G6T_* genes altered the expression of genes involved in regulation of shoot development, response to gibberellin, leaf development, auxin homeostasis, and other processes (Table S1). Based on our RNA-Seq data, we supposed that the long-root phenotype can be caused by the altered expression of auxin response genes, including *SMALL AUXIN-UP RNA*s (*SAUR*s), *Glycoside hydrolase 3* (*GH3*), and *Auxin Response Factor* (*ARF*) (Table S1). Among the DEGs in *StCLE4* overexpressing leaves, the *StIT1* gene was found. *StIT1* encodes a TCP transcription factor that determines tuber identity and interacts with the tuberization signal SP6A. The stolons of *it1* knockout mutants were converted into aerial shoot branches instead of swelling [8]. Based on our RNA-Seq analysis and the observed effects of *StCLE4* overexpression, it could be speculated that the StCLE4 peptide may initiate a signaling pathway that results in the inhibition of the *StIT1* gene expression. 

### 2.4. Yeast One-Hybrid Assay of Interaction between StNLPs and the Promoters of the StCLE4, StBEL5, StSP6A, and StIT1 Genes

We next performed yeast one-hybrid (Y1H) assay to identify the putative regulators of *StCLE4*. Previously, we found that the crucial regulator of the photoperiod-dependent tuberization StBEL5 did not bind to the DNA sequence corresponding to 500 bp upstream of the start codon of the *StCLE4* gene [4]. In this study, we performed an analysis of the direct binding of the key nitrate regulators NLPs to the promoter regions of *StCLE4*. NIN-like proteins (NLPs) are a family of transcription factors that play important roles in plant growth and development, as well as in plant–microbe interactions. The name “NIN-like” comes from the fact that these proteins are structurally similar to the Nodule Inception (NIN) protein, which is involved in the formation of root nodules in legume plants. One of the key functions of NLPs is the regulation of nitrate signaling and metabolism [9,10]. In Arabidopsis, the AtNLP6 and AtNLP7 proteins are crucial for the regulation of gene expression in response to nitrate [9,10,11]. Using NLP protein sequences from *A. thaliana*, *Medicago truncatula*, and *Solanum lucopersicum* as queries, three *StNLP* genes homologues to *AtNLP6* and *AtNLP7* were identified (*StNLP3.1* (Soltu.DM.08G004830.1), *StNLP3.2* (Soltu.DM.08G004830.2) and *StNLP5* (Soltu.DM.08G029670.1)) (Figure S4). Among the two variants of the *StNLP3* gene, *StNLP3.1* was selected for further investigation due to its higher expression levels in comparison to *StNLP3.2* (Table S1) (means of transcripts per million (TPM) values are 1722 and 80, respectively). The *StNLP5* gene is also highly expressed in all samples (mean of TPM = 2037). 

**Figure 4:**
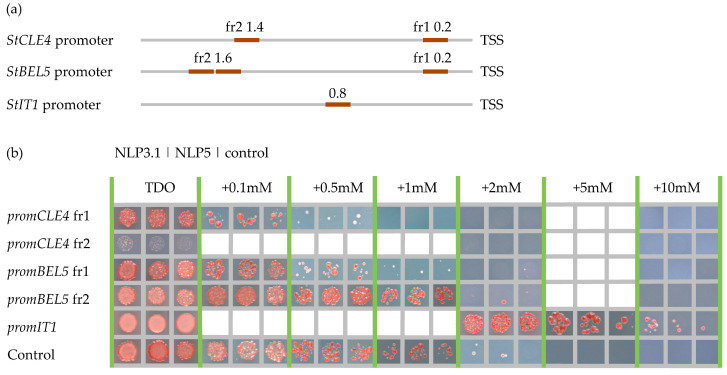
Interaction of StNLP3.1 and StNLP5 with *StCLE4*, *StBEL5*, and *StIT1* promoters. (**a**) Approximate locations of the NRE motifs (according to Nishida et al. [12] and Laffont et al. [13]) in *StCLE4*, *StBEL5*, and *StIT1* promoters. The numbers indicate the distance from the translational start site (TSS). (**b**) Yeast one-hybrid assays of the interaction of StNLP3.1 and StNLP5 with *StCLE4*, *StBEL5*, and *StIT1* promoters. Controls: empty pHISLEU or pDEST22 vectors.

NLPs are known to bind to nitrate-response elements (NREs) and activate the expression of nitrate-regulated genes in response to high nitrate levels [11]. We searched for the NREs in the promoter of the *StCLE4* gene, and in the promoters of major regulators of tuber development (*StBEL5*, *StSP6A*, and *StIT1* genes) using the motifs described in the studies by Nishida et al. [12] and Laffont et al. [13] In the promoters of the *StIT1*, *StCLE4*, and *StBEL5* genes, we found one, two, and three putative NLP-binding sites, respectively (Figure 4a, Table S2). Next, we checked whether these putative NREs motifs could be bound by StNLP3.1 and StNLP5 using yeast one-hybrid assay. We found that StNLP3.1 and StNLP5 bound to the promoter of the *StIT1* gene, whereas no interaction was detected for the *StCLE4* and *StBEL5* promoters (Figure 4b).

## 3. Discussion

Nitrogen is a crucial nutrient for the growth and development of potato plants, including tuber development. Previously, we found that the *StCLE4* gene was positively regulated by nitrogen supply and suggested that StCLE4 could be a negative regulator of tuberization. In this study, we found that *StCLE4*-overexpressing plants demonstrated a *wus*-like shoot phenotype, with the arrested growth of the SAM and subsequent recovery of shoot growth via the activation of axillary buds. This phenotype was similar to that described for overexpression of *CLV3*, *CLE2*, *-3*, *-4*, *-5*, *-6*, *-7*, *-9*, *-10*, *-11*, and *-13* genes in *A. thaliana* [6]. Like Arabidopsis plants with *AtCLE1-7* overexpression, potato plants overexpressing the *StCLE4* gene resumed growth after the initial SAM arrest. Furthermore, the StCLE4 is close to these AtCLE proteins according to phylogenetic analysis [4]. Besides this, *StCLE4* overexpression promoted root growth, and a similar effect was also observed for *AtCLE2* and *AtCLE4-7* overexpression [6]. However, opposite to *A. thaliana CLE1*, *-3*, *-4*, and *-7* genes, the *StCLE4* gene was positively regulated by nitrogen supply, like the *AtCLE2* and *AtCLE6* genes [3]. At the same time *AtCLE2* and *AtCLE6* promoters drive specific GUS staining in the cells at the junction between the primary root and the lateral roots [14], whereas *StCLE4* reporter expression was detected in the vascular tissues of the root. Therefore, StCLE4 is closely related to Arabidopsis AtCLE1-7 peptides according to phylogenetic analysis and functional studies; however, the regulation of *StCLE4* activity in potato is different from that described of the *AtCLE1-7* genes.

The overexpression of *StCLE4* not only affected shoot apex and root growth, but also promoted stolon conversion into branches. We performed RNA-Seq analysis of the leaves and roots of transgenic and control plants. At the same time, the downregulation of the *StIT1* gene was found in the leaves of *StCLE4*-overexpressing plants grown in N-deficient media. IT1 is a transcription factor that directly interacts with a mobile tuberigen, SP6A, and regulates tuber identity [8]. The stolons of *it1* knockout mutants were converted into branches instead of swelling [8]. Opposite to *it1* mutants, *StCLE4*-overexpressing plants produced tubers, but their weight was lower than that of the control plants. Therefore, we suppose that StCLE4 promotes the growth of stolons as shoots, similar to milder *it1* phenotype, and that the *StCLE4*-overexpressing phenotype could be explained by the downregulation of *IT1,* which in turn regulates stolon swelling. 

Based on the *StCLE4*-overexpressing phenotype, we supposed that the overexpression of *StCLE4_G6T_* should induce root shortening and increase tuber weight. However, we observed no changes in the growth of potato plants overexpressing *StCLE4_G6T_*, except for a decrease in tuber weight, compared to the control plants. This result indicates that while a Gly at position six is crucial for StCLE4 activity, StCLE4_G6T_ does not seem to function as an antagonistic peptide. Although Song et al. [5], Xu et al. [15], and Ren et al. [16] have demonstrated the effectiveness of the antagonistic peptide approach in several cases, its universal applicability remains to be determined. This approach was initially used for CLV3, CLE8, CLE19, and CLE22, and recently for the CLE25 peptide, but at the same time, for CLE45, it was shown that the antagonistic peptide acts as a weak native peptide rather than its antagonistic version [17]. Moreover, for some CLE peptides, it was demonstrated that the sixth amino acid in the CLE domain is not critical for their function, and its substitution does not produce an antagonistic peptide [17]. Our finding suggests that StCLE4_G6T_ may acts as a weak version of StCLE4 but not as an antagonistic peptide.

The NLPs transcription factors can respond to nitrate signaling, regulate gene expression, and participate in N absorption and utilization. Previously, it was shown that NLP can regulate the expression of the *CLE* genes [13,18,19], and therefore, we assumed that StNLP3 and StNLP5 can bind to the promoter region of *StCLE4*. However, we did not detect this interaction in the yeast one-hybrid system. Moreover, previously, we also did not find any interaction of photoperiod-dependent tuberization factor StBEL5 and the promoter region of the *StCLE4* gene [4], and therefore, we still do not know which transcription factor regulates *StCLE4* gene expression. However, we found that StNLP3 and StNLP5 bound to the promoter of *StIT1*. Previously, it was shown that N plays an important role in regulating tuberization [20], and based on our data, we speculate that the StCLE4 peptide, as well as the StNLP3 and StNLP5 transcription factors, may act on the N-depended tuberization pathway and regulate the expression of *StIT1*, however, further investigations are necessary to study the mechanisms of such regulation in more detail. Further research on the mechanisms of *CLE* genes regulation and their interactions with nitrogen signaling pathways will be crucial for understanding how plants respond to changes in nitrogen availability and for developing strategies to improve crop productivity.

## 4. Materials and Methods

### 4.1. Plant Materials and Growth Conditions

Tubers of the *Solanum tuberosum* cultivar Desirée was obtained from the stock of N. I. Vavilov All-Russian Institute of Plant Genetic Resources (Saint-Petersburg, Russia). The plants were propagated by two-node stem cuttings and were cultivated in vitro on a Murashige and Skoog (MS) medium containing 0.8% (*w*/*v*) agar and 1% sucrose under long-day conditions (16 h light/8 h dark) at 22 °C. N-depleted or N-rich medium was prepared as described in [21]. The N-rich medium contains 10 mM NH_4_Cl and 10 mM KNO_3_ as sources of nitrogen and potassium, respectively. The other elements are present at half-strength concentrations. The N-depleted medium does not contain NH_4_Cl and KNO_3_ but instead has 10 mM KCl added.

For the investigation of the effects of *StCLE4* and *StCLE4_G6T_* overexpression on root growth, aeroponic nutrient medium without nitrate was used [22]. Seven-day-old stem cuttings were transferred into aeroponic system in a controlled greenhouse (16 h light/8 h dark and 22 °C) and were grown for four weeks. The root length was measured using ImageJ v. 1.52r [23] software, and Tukey’s test was used to compare the means of three groups. Stem cuttings were also transferred to pots (9 × 9 × 9 cm) with vermiculite and grown in a growth chamber for three months (16 h light/8 h dark and 22 °C for the first two months; 8 h light/16 h dark and 22 °C for the induction of tuber development). Twice a month plants were supplied with ¼ MS media without sucrose. Tubers, obtained from plants growing in the vermiculite, were planted in the field (Leningrad Region, Russia) in June 2023 and were grown for 2.5 months. The tuber weight of the plants grown in the field was measured, and Tukey’s test was used to compare the means of three groups. Boxplots were generated using boxplot package in R [24].

### 4.2. Constructs

The primers used for PCR are listed in Table S3. Polymerase chain reaction (PCR) with Phusion High-Fidelity DNA Polymerase (Thermo Scientific, Waltham, MA, USA) was used to amplify coding sequences or predicted promoter fragments of genes from the whole-plant DNA of *Solanum tuberosum* L. cv Desiree. PCR fragments were extracted from an agarose gel using a Cleanup Mini Kit (Evrogen, Moscow, Russia) and cloned into the entry vector pDONR207. Then, coding sequences were cloned into the vector pMDC32 [25] under the CaMV 35S promoter, via Gateway technology, according to the manufacturer’s instructions (Invitrogen, Waltham, MA, USA). Promoter sequences for GUS or GFP reporter analysis were cloned into the vector pBGWFS7 [26] or pHm43GW (Invitrogen, USA). For the yeast one-hybrid assay, the promoter fragments of the *StCLE4*, *StSP6A*, and *StIT1* genes (Table S2) were cloned into the pHISLEU2GW vector (kindly provided by Dr. Rogers from Cardiff University). The coding sequences of *StNLP3.1* and *StNLP5* were introduced into pDEST22 (Invitrogen, USA). The resulting plasmids were transformed into Top10 *Escherichia coli* Competent Cells. Plasmid DNA was isolated using a Plasmid Miniprep Kit (Evrogen, Russia). Each plasmid was checked by sequencing. The resulting binary vectors were transferred to the *Agrobacterium rhizogenes* strain Arqua or *Agrobacterium tumefaciens* strain AGL1. Agrobacterium cultures were grown overnight on a shaking incubator at 28–30 °C in Luria–Bertani supplemented with antibiotics. 

### 4.3. Transformation of Potato

For hairy potato root transformation with the *promStCLE4::GUS* construct, stems were wounded and inoculated with the *A. rhizogenes* and then co-cultivated on Murashige and Skoog (MS) media with 10 g/L of sucrose for 2 days. Transgenic hairy roots were grown in MS medium supplemented with cefotaxime (500 mg/L) to prevent *Agrobacterium* overgrowth.

Transgenic *S. tuberosum* plants containing *prom35S::StCLE4* and *prom35S::CLE4_G6T_* constructs were generated by *Agrobacterium tumefaciens*-mediated transformation mainly as described previously [27,28,29]. The wounded leaf explants were incubated with agrobacteria in liquid MS medium with 16 g/L glucose for 2 days. Then, the explants were moved to solidified MS medium (8 g/L agar) that induces callus growth (16 g/L glucose; 500 mg/L cefotaxime; 3 mg/L hygromycin; 5 mg/L NAA; 0.1 mg/L BAP) and were incubated for 7–8 d under LD at 27 °C. The explants were later transferred to a shoot-regeneration MS medium (16 g/L glucose; 500 mg/L cefotaxime; 3 mg/L hygromycin; 1 mg/L BAP; 0.1 mg/L GA) and were repeatedly transferred to fresh medium with the same composition every 10 days until shoots developed on the calli. The resulting 3 cm shoots were then transferred to MS medium (10 g/L sucrose; 500 mg/L cefotaxime) for rooting.

### 4.4. Histochemical Assays

β-Glucuronidase (GUS) staining was performed as described in [30]. Incubation periods ranged from 2 to 16 h following vacuum infiltration. Roots were imaged using a ZEISS SteREO Discovery.V12 microscope. 

### 4.5. RNA Isolation and RNA-Seq

For RNA-Seq analyses, total RNA was extracted from samples using a RNeasy Plant Mini Kit (Qiagen, Germantown, MD, USA). The enrichment of mRNA was performed using a NEBNext^®^ Poly(A) mRNA Magnetic Isolation Module (New England Biolabs, Ipswich, MA, USA). The libraries were prepared from three to four biological replicates for each of the transgenic plants and conditions for a total of 30 transcriptome libraries using a NEBNext^®^ Ultra™ II Directional kit (New England Biolabs, USA). For sequencing, a HiSeq PE Rapid Cluster Kit v2 and HiSeq Rapid SBS Kit v2 were used. Manufacturer’s instructions were used for all procedures. The libraries were sequenced in the Centre for Molecular and Cell Technologies of Saint Petersburg State University Research Park using the Illumina HiSeq 2500 sequencing platform. 

The quality control of the reads was performed using FASTQC v. 0.11.9 (http://www.bioinformatics.babraham.ac.uk/projects/fastqc/ (accessed on 1 July 2022)). FastQC reports were aggregated using MultiQC v. 1.14 [31]. The trimmomatic v. 0.39 program [32] was used to trim reads from technical artifacts. HISAT2 v. 2.2.1 [33] was used as the alignment program for mapping reads to a doubled monoploid potato DM 1-3 516 R44 genome v. 6.1. BAM files were sorted using samtools v. 1.13. The quantitation of RNA-Seq data was performed using StringTie v. 2.2.1 [34]. The *StCLE* and *StCEP* (C-terminally encoded peptide) [35] genes that have not been annotated previously as coding sequences were manually included in annotation. Differential gene expression analysis was performed using DeSeq2 v. 1.40.2 [36] in R [24]. The Cut-off criteria for DEGs significance was padj < 0.05 and the absolute value of the log2 fold change >1. Volcano plots were generated using Enhanced Volcano R package [24,37].

### 4.6. Yeast One-Hybrid Assay 

The 2.5 kb sequences upstream of the start codon were used for the promoter analysis. Putative NRE motifs were identified using sequences revised in several recent studies [12,13] and MAST algorithms available on the MEME suite server (version 5.1.0, https://meme-suite.org/meme/tools/meme (accessed on 1 September 2023)). The transformation of the *Saccharomyces cerevisiae* strain Y2HGold (Clontech) was performed as described in [38]. A yeast one-hybrid assay was performed as described in [39].

### 4.7. Identification and Phylogenetic Analysis of the NLP proteins

To identify NLP proteins in potato, BLASTP analyses were performed using all the *Arabidopsis thaliana* NLP*s* as queries against proteins of the *S. tuberosum* group Phureja DM1-3 516 R44. The amino acid sequences of the NLP proteins from *Arabidopsis thaliana*, *Medicago truncatula*, *Solanum lycopersicum*, *Oryza sativa*, *Lotus japonicas*, and *Solanum tuberosum* were aligned using the muscle algorithm in MEGA7 software. A phylogenetic tree with 1000 bootstrap replicates was constructed using the MEGA7 neighbor-joining method with default parameters [40].

## Data Availability

All sequence reads of the RNA-Seq analysis have been deposited at the National Center for Biotechnology Information database under BioProject accession number PRJNA1007918.

## Supplementary Materials

The following supporting information can be downloaded at: https://www.mdpi.com/article/10.3390/plants12193468/s1, Figure S1: The phenotype of *StCLE4_G6T_*-overexpressing plants that were grown in growth chamber; Figure S2: The phenotype of *StCLE4* (*StCLE4-oe*) and *StCLE4_G6T_* (*StCLE4_G6T_-oe*)-overexpressing plants, and control plants that were grown in the field; Figure S3: The volcano plots of DEGs between *StCLE4oe* and control plants; Figure S4: Phylogenetic tree of *Arabidopsis thaliana*, *Medicago truncatula*, *Solanum lycopersicum*, *Oryza sativa*, *Lotus japonicas*, and *Solanum tuberosum* NLP protein families; Table S1: Differentially expressed genes (DEGs) in the leaves and roots of control and transgenic plants; Table S2: Potential NRE sites in the promoter regions of *StCLE4*, *StBEL5*, *StIT1*, which were used for yeast-one-hybrid analyses; Table S3: List of primers.
