# Supplementary material for: Nitrogen-Activated CLV3/ESR-Related 4 (CLE4) Regulates Shoot, Root, and Stolon Growth in Potato
Source: Plants (Basel). 2023 Oct 3;12(19):3468. doi: 10.3390/plants12193468 (PMC10574742; doi:10.3390/plants12193468)

Supplementary Figures

Supplementary Figure S1. The phenotype of *StCLE4*$_{G6T}$ (*StCLE4* $_{G6T}$ *-oe)* overexpressing plant. (a) Plants were grown in vermiculite in the growth chamber for first 2 month under long day and three weeks under short days (b) Apexes of plants that were grown in vermiculite in the growth chamber for three weeks.

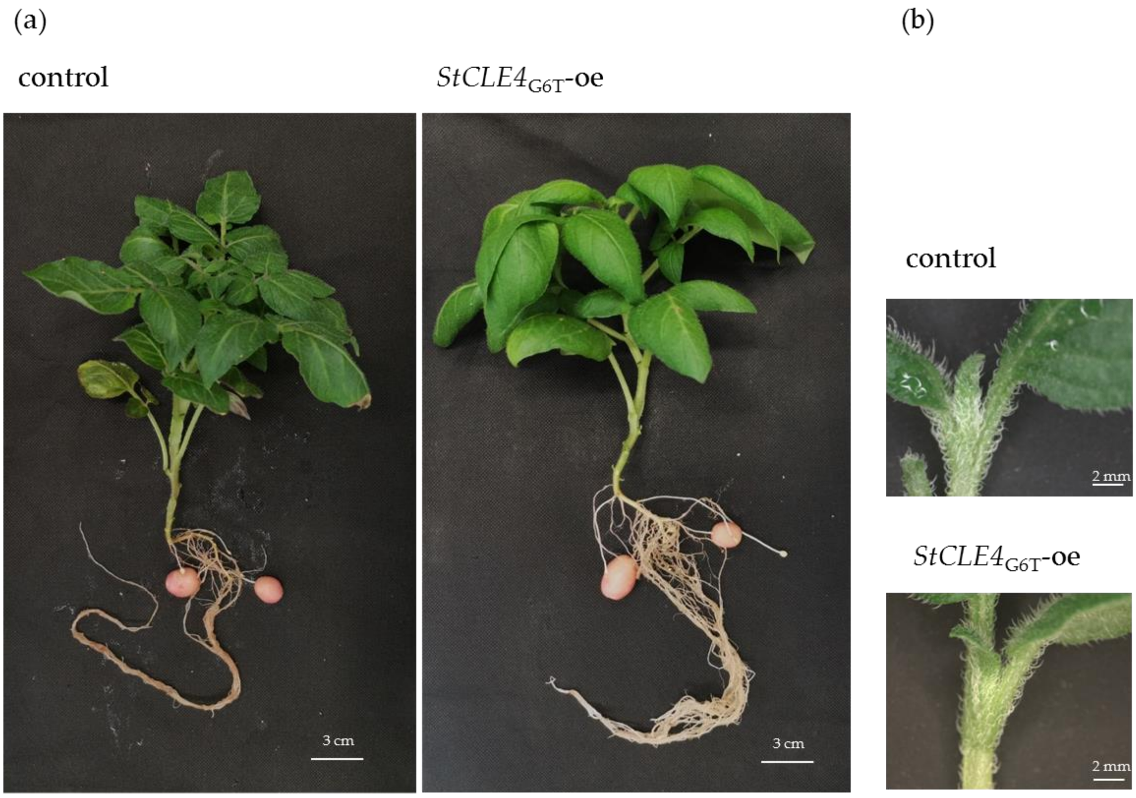

Supplementary Figure S2. The phenotype of *StCLE4* (*StCLE4-oe*) and *StCLE4*$_{G6T}$ (*StCLE4*$_{G6T}$*-oe*) overexpressing plants, and control plants that were grown in the field for 2.5 months. Red arrowheads indicate stolons that converted into branches.

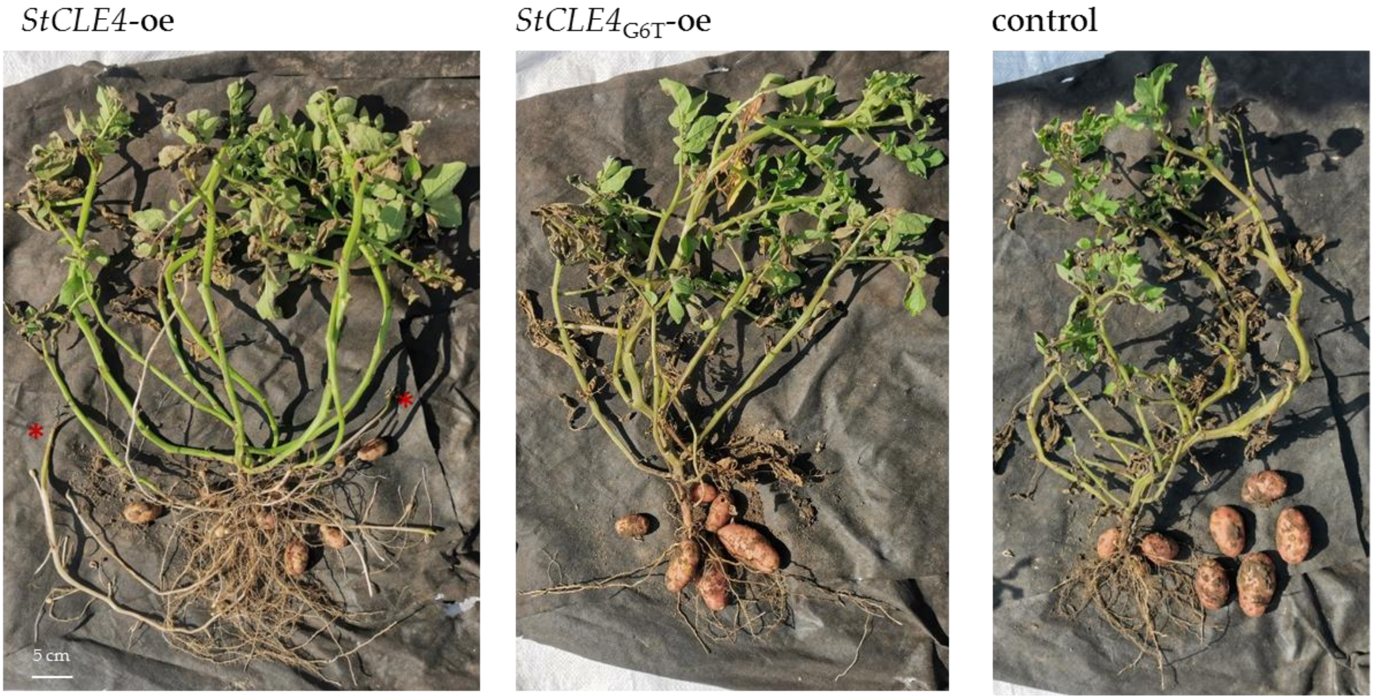

Supplementary Figure S3. The volcano plots of DEGs between *StCLE4oe* and control. The top 10 upregulated and downregulated genes are labeled.

(a) DEGs in leaves

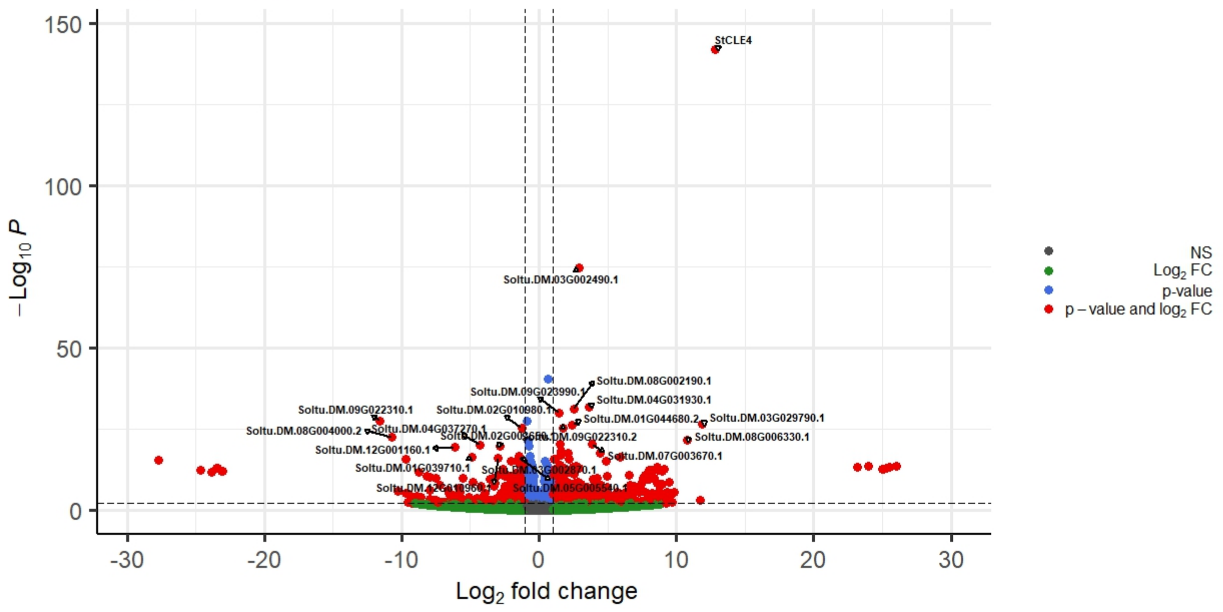

(b) DEGs in roots

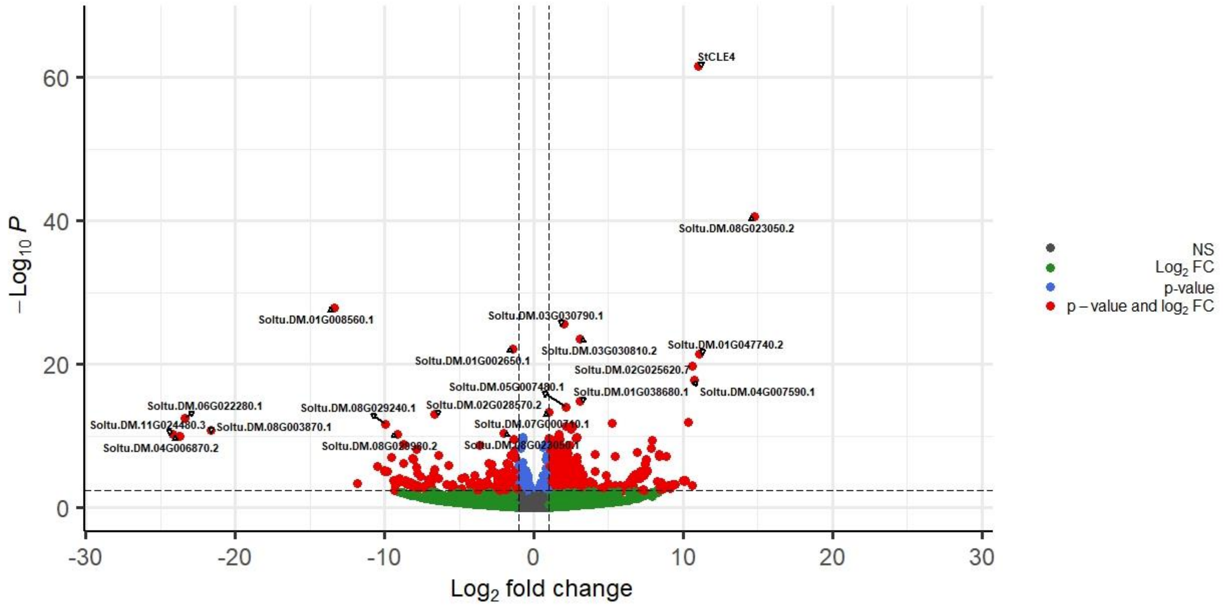

Supplementary Figure S4. Phylogenetic tree of *Arabidopsis thaliana*, *Medicago truncatula*, *Solanum lycopersicum*, *Oryza sativa*, *Lotus japonicas*, and *Solanum tuberosum* (red) NLP protein families.

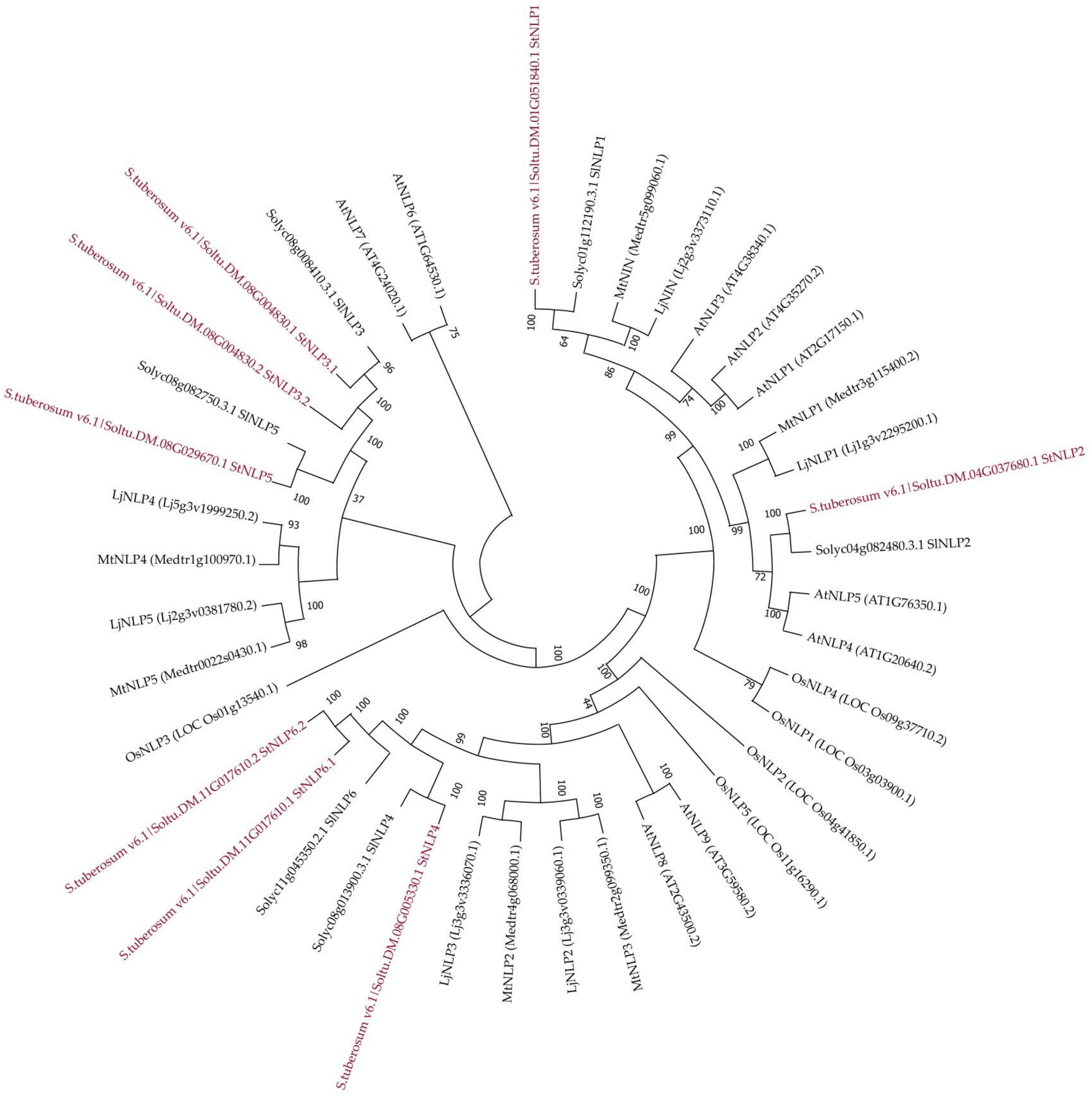

Supplement: Supplementary file 1 [file plants-12-03468-s001.zip › Supplementary Figures.pdf]
