# Supplementary material for: Nitrogen-Activated CLV3/ESR-Related 4 (CLE4) Regulates Shoot, Root, and Stolon Growth in Potato
Source: Plants (Basel). 2023 Oct 3;12(19):3468. doi: 10.3390/plants12193468 (PMC10574742; doi:10.3390/plants12193468)
Supplement: Supplementary file 1 [file plants-12-03468-s001.zip › Table S2.pdf]

Table S2: Potential NRE sites in the promoter regions of *StCLE4*, *StBEL5*, *StIT1*, which were used for yeast-one-hybrid analyses. Ref- reference genome v6.1 for the doubled monoploid potato DM 1-3 516 R44. Highline sequences have alterations in Desiree genome.

>pCLE4_region_1_ref

TAACACTCCTATAAAGCAAGAACTCATGAAATTAATAAGCATACAAATAAATATTGATAATATA
GATATCCATATAATTATTCGTCTGAATATTTATCTTTTAGCGAATAATATATATCCATTTGTATTCA
TTTTTTAAAGAATCGATTATTCGATCACTTTAAATAGTTACTCATTTTTTATATCAAAAAGGTGCAT
TAAAAAAATTCAGGTTGACAACTTGAATAGCTAGTACTAATATAATTTCTCTTTGATCAGGTCAA
ATGGCCTAAAGGGTATAATACAAAAAAAGTTTGTTTTAAACAAGTTATCAAATCACTATTACACT
TTTCGTTCACATTTTTAGCACTTATCTTATTTGATTGTGAACCCACCTTGTCAATAATATTATATAC
TATGAATATTTAGGACTATATATATAGTTCAATAATCACAACACAATATAACTCTCTTCATTTCAT
GCAAACCTTAGATATATATCATTAATTATTCAAGAATTA

>pCLE4_ region_1_Desiree

TAACACTCCTATAAAGCAAGAACTTATGAAATTAATACACATACAAATAAGTATTGATAATATA
ACTATTCATATAATTATTCGTCTGAATATTTATTTTTTAGTGAATAATATATATCCATTTTTCGTATT
CATTTTTAAAGAATCAATTATCCAACTCATATCTAATGGATCAGTTTAAATAGTTACTCATTTTTA
AAACCATTTTGCTAGGCTGCATACCAAAAAGGTGCATTAAAAAAATTCAGGTTGACAACTTGAA
TGGCTAGTACTAATATAATTTCTCTTTGATCAGGTCAAATGGCCTAAAAAGTAGAATACAAAAAA
TGTTTGTTTTAAACAAGTTATCAAATCACTATTACACTTTTCTTTCTCATTTTTAGCACTTTATCTTA
TTTGATTGTGAACCCCCCTTGTCAATAATATTATATACTATGAATATTTAGGACTATATATATAGTT
CAATAATCACAACACAATATAACTATCTTCATTTCATGCAAACCTTAGATATATATCATTAATTAT
TCAAGAATTA

>pBEL5_ region_2_ref

CATTGTACCATTGCGTCACGCTAATATAAGTAAACTTTTAAGAAGAAAGAGAGACTTTCTTACTC
ATCACATTACTTAGTCAATTTTTTTTTAAAGAAAAATCTCTTTTCCTATTTAGCAACTATTTGATTC
CAAATTTCCACATAAATCTCAAATATTACAATGTTAAAAAACATTTTGAAGTATTTGATTAAAAG
TTTTTTTTTTTTTTTACTTTCTTAAATTACGTGTCAAATTAAAATAGTACAAACAAATTAAAATTGG
AGAGTACTAGAAAATAAGAAATTGACGATGTATAAAATTTTGTAGCTAAATAAAAAAAATTCTA
TACTAATTTTATTTAGCTACAAAATAAAGATGAATTATAAGAAAATCCAATTAGCTTGGAACGTT
TTATAAATAAATTAACTATGAATTGTCCCATAAATTCTATAGGAGAACAAAAATGTTGCCCCTTA
CATCACTCCACAATTTACCGAAACAAAAAAAAAGTAAATACGCACCTTTCATCTTTCAAGT

>pBEL5_ region_2_Desiree

CATTGTACCATTGCGTCACGCTAATATAAGTAAACTTTTAAGAAGAAAGAGAGACTTTCTTACTC
ATCACATTTACTTAGTCAATTTTTTTTTAAAGAAAAATCTCTTTTCCTATTTAGCAACTATTTGATT
CCAAATTTCCACATAAATCTCAAATATTACAATATTAAAAAATATTTTGAAGCATTTGATTAAAA
GATTTAATTTTTTTTACTTTCTTAAATTATGTGTCAAATCAAAATAATACAAACAAATTAAAATTG
GAGAGTACTAGAAAATAAGAAATTGACGATGTATAAAAATTTGTAGCTAAATTAAAATATTCTA
TACTAATTTTATTTAGCTACAAAATAAAGATAAATTATAAGAAAATTCAATTAGCTCGGAACGTT
TTATAAATAAATTAGCTATGAATTGTCCATAAATTCTATAGGAGAAGAAAAATGTTGCCCCTTAC
ATCACTCCACAATCTACCGAAACAAAAAAAAGTAAATACGCACCTTTCATCTTTCAAGT

>pBEL5_ region_1_ref

AGATGAAACCAATAAAGATTAGCAGTACATTTTTCTTCAATTGGTTTCACAATTAATCACTTTACT
AATCGTTATCTCAGAAAGTCTATTTTTAATGTTATCTTCGTTTACTTTTACTTATTAAATTTACTAAA
AAGAAATTTAAGAAGTCATGTTTTTCCTTCAACGTTAATTATTTATTTTTCAATTTTTTTTCCATTAC
CAAAAGATATACATCCATTAGTAGGAGTATCGTTATAAAATGTTTATGTCAATCATTTTTTTTTAA
AGGATCCGTAAAAATTAAAATTAAACAAGTAAAAATAAATGGTGGAAGTATATATATTCAATAC
ACAAAGCAGTGTACTTTCATTTCTTGG

>pBEL5_ region_1_Desiree

AGATGAAACCAATAAAGATTAGCAGTACATTTTTCTTCAATTGGTTTCACAATTAATCACTTTACT
AATCGTTATTATCTCAGAACCTTCGTTTACTTTTACTTATTAAATTTACTAAAAAGAAATTTAAGA
AATCATGTTTTACCTTCAACGTTAATTATTTATTTTTCAATTTTTTTTCCATTACCAAAAGATATACA
TCCATTAGTAGGAGTATCGTTATAAAATGTTTATGTCAATCATTTTTTTTTTAAAGGATCCGTAAA
AATTAAAATTAAACAAGTAAAAAAAAAATGGTGGAAGTACATATATTCAATACACAAAGCAGTGT
ACTTTCATTTCTTGG

>pIT1_Ref

GATCTGCCACATAACATAACAAATGGTTTCAAACGGAACATATGACTCTTAATAAAAGGTGGAG
AAGGGTTGAAAACTTTACTAAATTTAACAAGATCTTAGGTGTGTATTTGACTCATGTTGCAAGATT
GAAGTGTATTTAAGTTCAGTTAATCAAGTAAAGAGATATTTTTAAGGTTGTCAATATATAATTCGG
AGATAAAACTAGCCTTTTGTTTCATTTGTTGAGTTTAAAATTAAGTTGATATGAAACAACAAATA
AATTGTGATCCCAAGTTTTATATGAGCA

> pIT1_Desiree

GATCTGCCACATAACATAACAAATGGTTTCAAACGGAACATATGACTCTTAATAAAAGGTGGAG
AAGGGTTGAAAACTTTACTAAATTTAACAAGATCTTAGGTGTGTATTTGACTCATGTTGCAAGATT
GAAGTGTATTTAAGTTCAGTTAATCAAGTAAATAGATATTTTTAAGGTTGTCAATATATAATTCGG
AGATAAAACTAGCCTTTTGTTTCATTTGTTGAGTTTAAAATTAAGTTGATATGAAACAACAAATA
AATTGTGATCCCAAGTTTTATATGAGCA
