# Supplementary material for: Nitrogen-Activated CLV3/ESR-Related 4 (CLE4) Regulates Shoot, Root, and Stolon Growth in Potato
Source: Plants (Basel). 2023 Oct 3;12(19):3468. doi: 10.3390/plants12193468 (PMC10574742; doi:10.3390/plants12193468)
Supplement: Supplementary file 1 [file plants-12-03468-s001.zip › Table S3.pdf]

Table S3: List of primers. AttB sites for GateWay cloning are highlined.

promStIT1	F	GGGGACAAGTTTGTACAAAAAAGCAGGCT GATCTGCCACATAACATAACAAATG	Y1H
promStIT1	R	GGGGACCACTTTGTACAAGAAAGCTGGGT TGCTCATATAAAACTTGGGATCAC	Y1H
promStBEL5 region_1	F	GGGGACAAGTTTGTACAAAAAAGCAGGCT AGATGAAACCAATAAAGATTAGCA	Y1H
promStBEL5 region_1	R	GGGGACCACTTTGTACAAGAAAGCTGGGT CCAAGAAATGAAAGTACACTGC	Y1H
promStBEL5 region_2	F	GGGGACAAGTTTGTACAAAAAAGCAGGCT CATTGTACCATTGCGTCACG	Y1H
promStBEL5 region_2	R	GGGGACCACTTTGTACAAGAAAGCTGGGT ACTTGAAAGATGAAAGGTGCGT	Y1H
StNLP5 cds	F	GGGGACAAGTTTGTACAAAAAAGCAGGCT ATGAATTTCATTTTCCGTTCAAAGCCT	Y1H
StNLP5 cds	R	GGGGACCACTTTGTACAAGAAAGCTGGGT TCATTCACCTGAGCTCTCACAGG	Y1H
StNLP3 cds	F	GGGGACAAGTTTGTACAAAAAAGCAGGCT ATGTCGGAACCGGAAGGAGG	Y1H
StNLP3 cds	R	GGGGACCACTTTGTACAAGAAAGCTGGGT TCATTTACCTGAGCTCTCACAGGAG	Y1H
promStCLE4 region_1	F	GGGGACAAGTTTGTACAAAAAAGCAGGCT TAACACTCCTATAAAGCAAGAACTC	Y1H
promStCLE4 region_1	R	GGGGACCACTTTGTACAAGAAAGCTGGGT TAATTCTTGAATAATTAATGATATATATCTAAGGTT	Y1H and GUS
promStCLE4 region_2	F	GGGGACAAGTTTGTACAAAAAAGCAGGCT GCAGCCGACATCAAATAGAAG	Y1H
promStCLE4 region_2	R	GGGGACCACTTTGTACAAGAAAGCTGGGT GGACCTTAGTTTAGTTAAAATGTG	Y1H
StCLE4 cds	F	GGGGACAAGTTTGTACAAAAAAGCAGGCT ATGGCTAGTGCTTCTAGGTTTTTGTT	Overexpr.
StCLE4 cds	R	GGGGACCACTTTGTACAAGAAAGCTGGGTC TAGTGATGTCGAGGATCAGGTCC	Overexpr.
StCLE4 G6T cds	R	GGGGACCACTTTGTACAAGAAAGCTGGGTC CTAGTGATGTCGAGGATCAGGTGTGTCAGGAC	Overexpr.
promStCLE4	F	GGGGACAAGTTTGTACAAAAAAGCAGGCT CTCATAAATCCCTAACATAGGTAAATAAT	GUS
